# Supplementary material for: Exploiting macrophage autophagy-lysosomal biogenesis as a therapy for atherosclerosis
Source: Nat Commun. 2017 Jun 7;8:15750. doi: 10.1038/ncomms15750 (PMC5467270; doi:10.1038/ncomms15750)
Supplement: Supplementary Information — Supplementary Figures and Supplementary Tables. [file ncomms15750-s1.pdf]

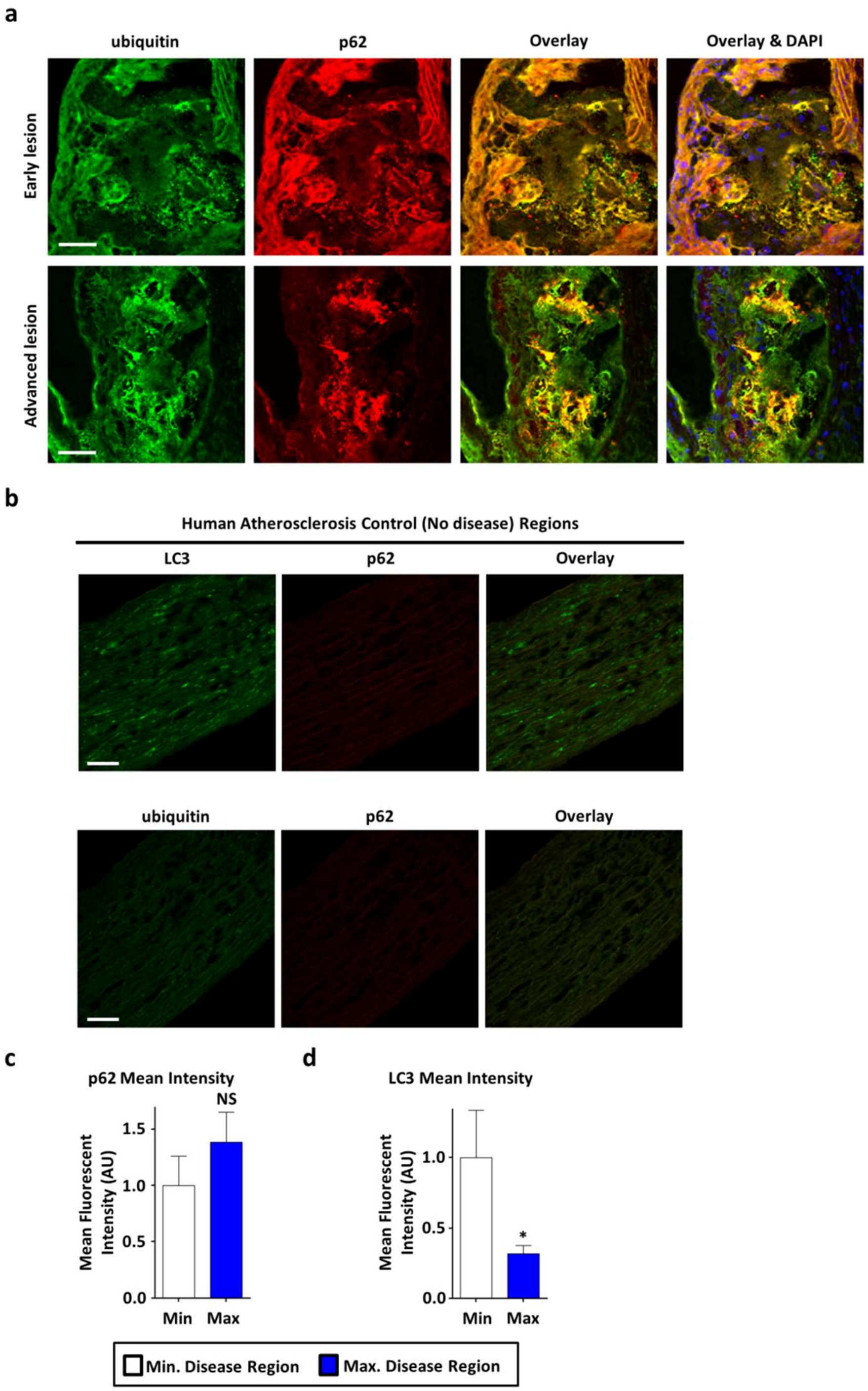

Supplementary Figure 1. (a) Representative immunofluorescence images of early-stage and more advanced mouse atherosclerotic (ApoE-KO) aortic roots co-stained with antibodies against polyubiquitin (FK-1) and p62 (scale bar: 50 μm). **(b)** Representative immunofluorescence images of non-diseased (control) areas of human carotid endarterectomy

specimens stained with LC3 and p62 (top) or polyubiquitin and p62 (bottom) (scale bar: 100 μm). **(c, d)** Mean intensity for LC3 and p62 staining were analyzed in human atherosclerosis samples (n=8) from minimally (min.) and maximally (max.) diseased regions. For all graphs, data are presented as mean ±SEM. *$P < 0.05$, NS=not significant, two-tailed unpaired t-test.

a

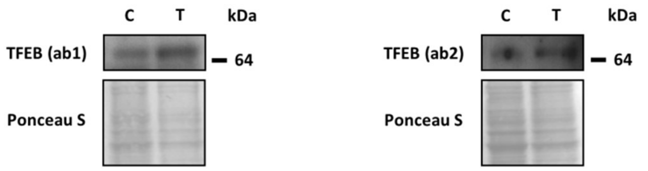

b-g

Please see next 3 pages for the serial montage images of the live-imaging experiments.

h

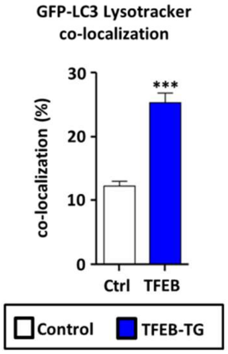

Supplementary Figure 2. (a) Western blot analysis of TFEB using two different antibodies from either control (C) or TFEB-TG (T) macrophages. Ponceau S staining is shown as loading control. **(b-e)** Representative 20-minute montage of live imaged **(b)** control GFP-LC3 macrophages in DMEM, **(c)** dual TFEB transgenic (TFEB-TG)/GFP-LC3 macrophages in DMEM, **(d)** control GFP-LC3 macrophages in 200 nM bafilomycin, **(e)** and dual TFEB-TG/GFP-LC3 macrophages in 200 nM bafilomycin (scale bar: 10 µm). **(f-g)** Representative 10-minute montage of live imaged **(f)** control GFP-LC3 or **(g)** TFEB-TG/GFP-LC3 macrophages after lysotracker-red staining (scale bar: 5 µm). **(h)** Compiled data representing GFP-LC3 and Lysotracker co-localization in TFEB-TG macrophages during ten minutes long live imaging experiment as shown in Figure 2g (n≥10 cells for each treatment). Data are presented as mean ±SEM. ***P < 0.001, two-tailed unpaired t-test.

b

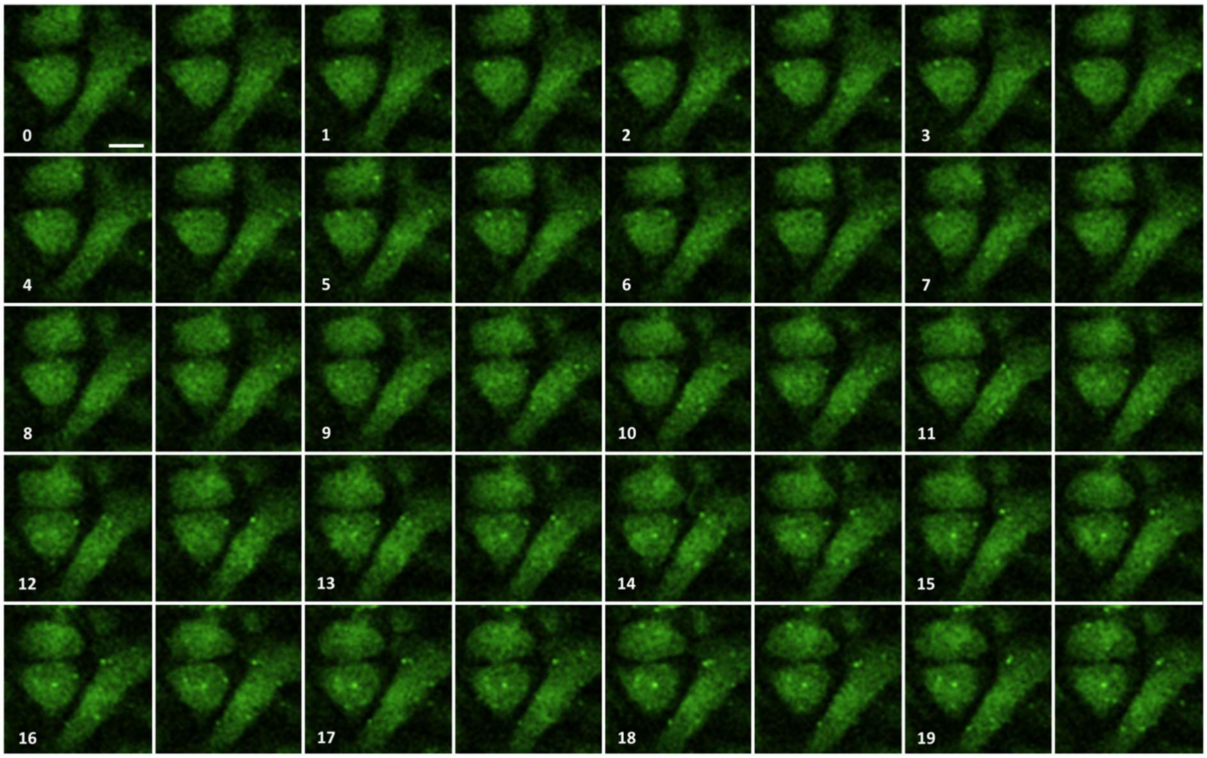

Control GFP-LC3 peritoneal macrophage in DMEM for 20 minutes (Frame/30 seconds)

c

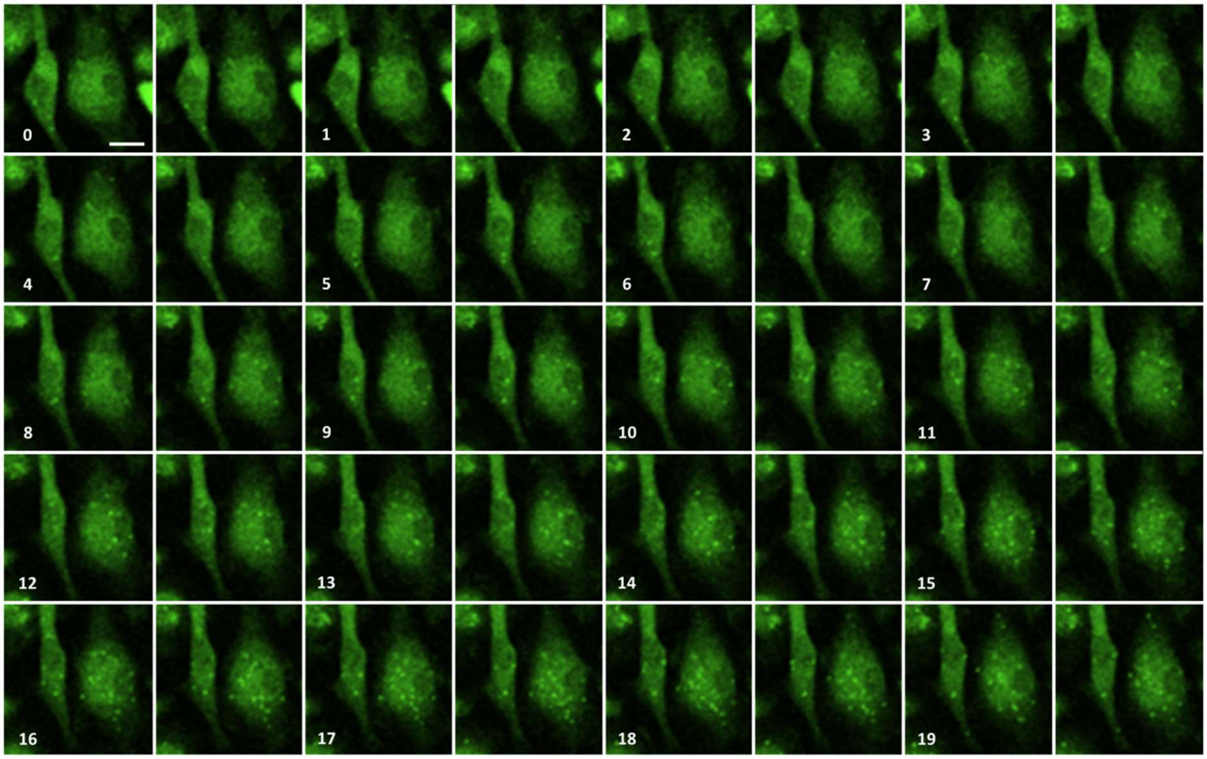

TFEB-TG GFP-LC3 peritoneal macrophage in DMEM for 20 minutes (Frame/30 seconds)

d

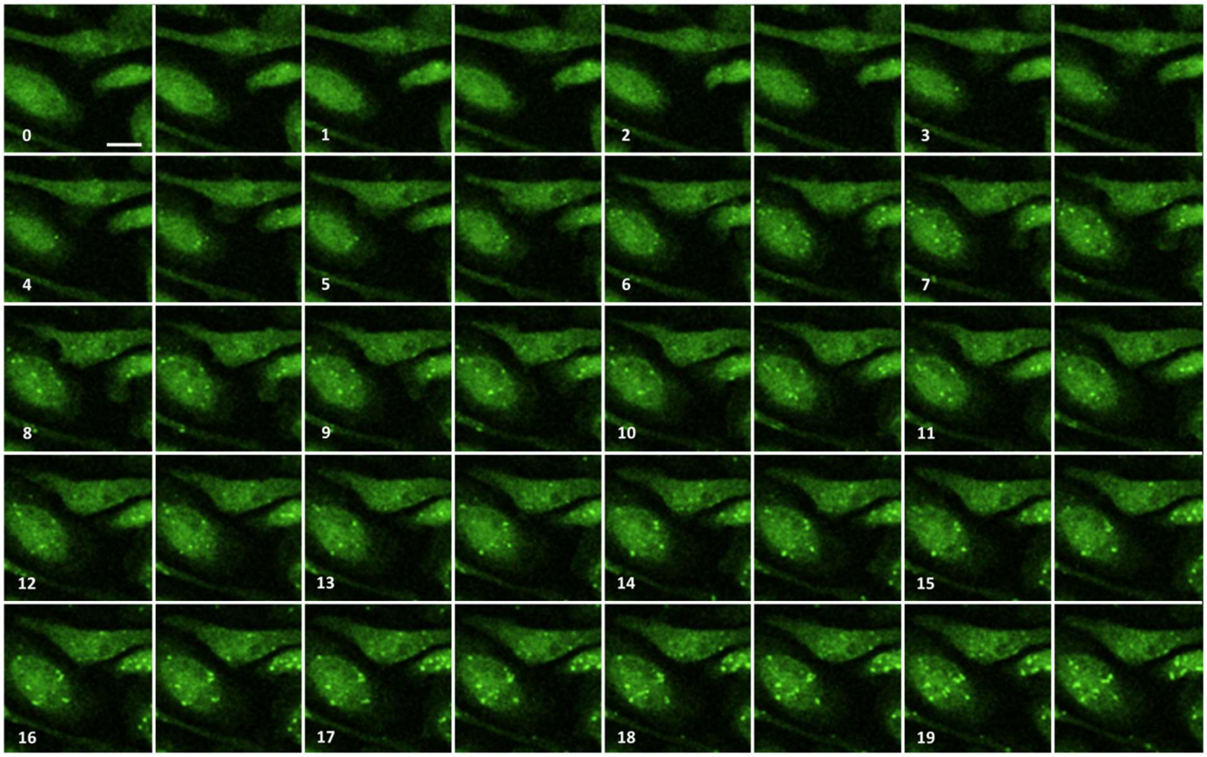

Control GFP-LC3 peritoneal macrophage in Bafilomycin for 20 minutes (Frame/30 seconds)

e

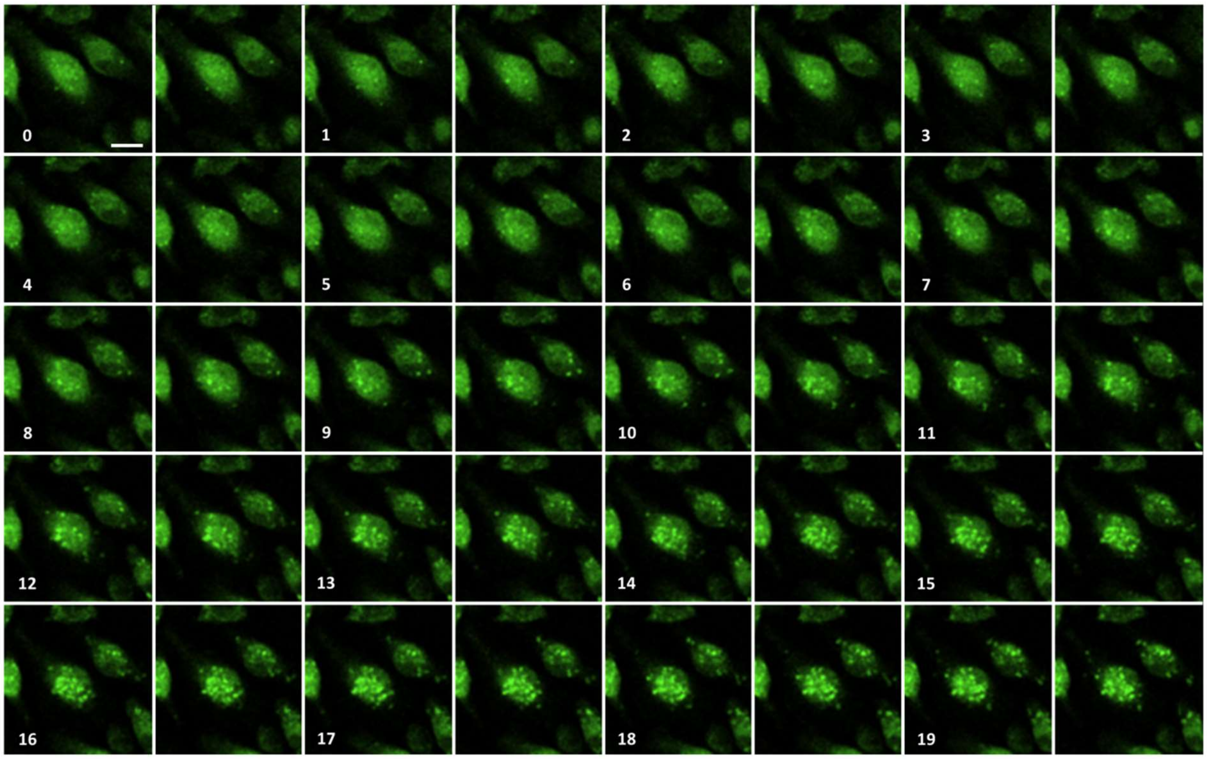

TFEB-TG GFP-LC3 peritoneal macrophage in Bafilomycin for 20 minutes (Frame/30 seconds)

f

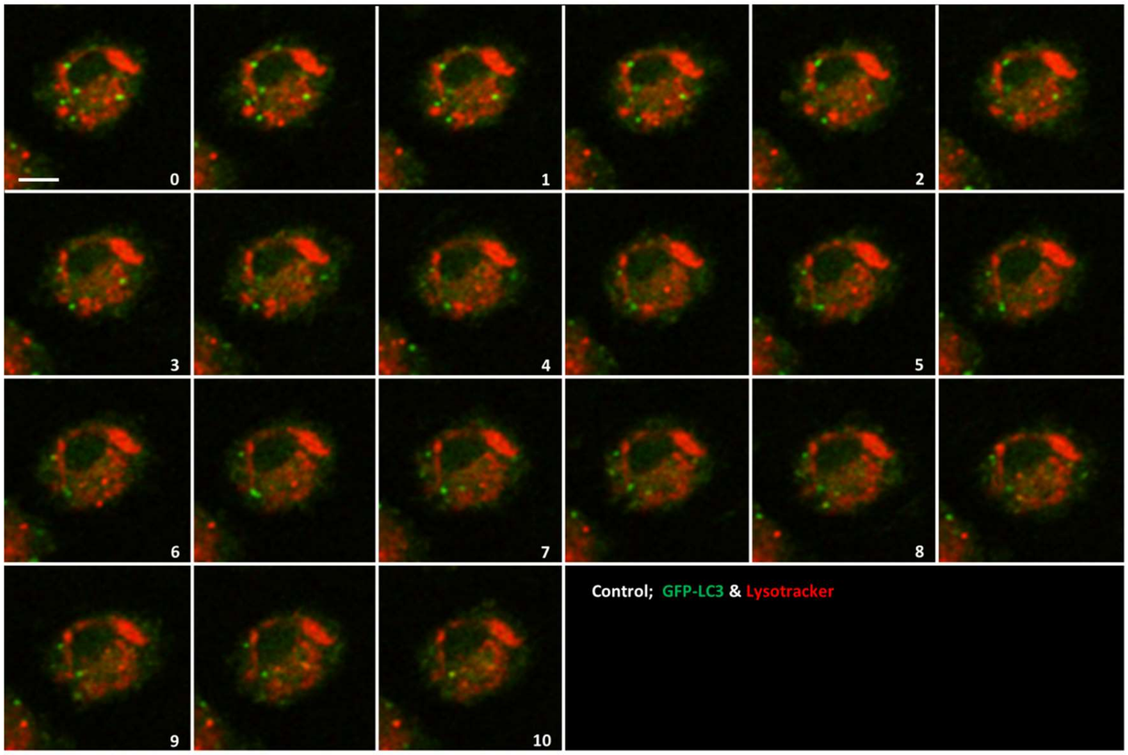

Lysotracker stained Control GFP-LC3 peritoneal macrophage in DMEM for 10 minutes (Frame/30 seconds)

g

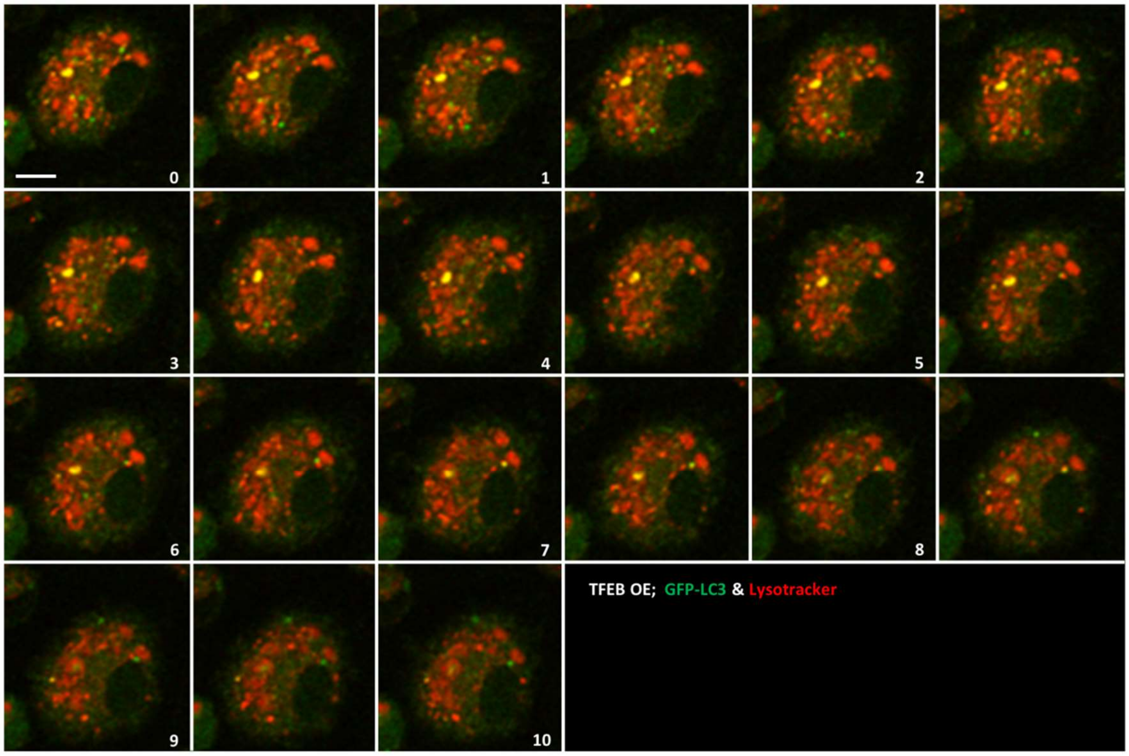

Lysotracker stained TFEB-TG GFP-LC3 peritoneal macrophage in DMEM for 10 minutes (Frame/30 seconds)

a

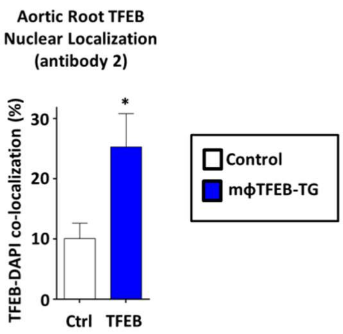

Supplementary Figure 3. (a) Quantification of the TFEB co-localization with the nuclear marker DAPI using a second TFEB antibody akin to Figure 3c (n=4-5 mice per group). Data are presented as mean ±SEM. *P < 0.05, two-tailed unpaired t-test.

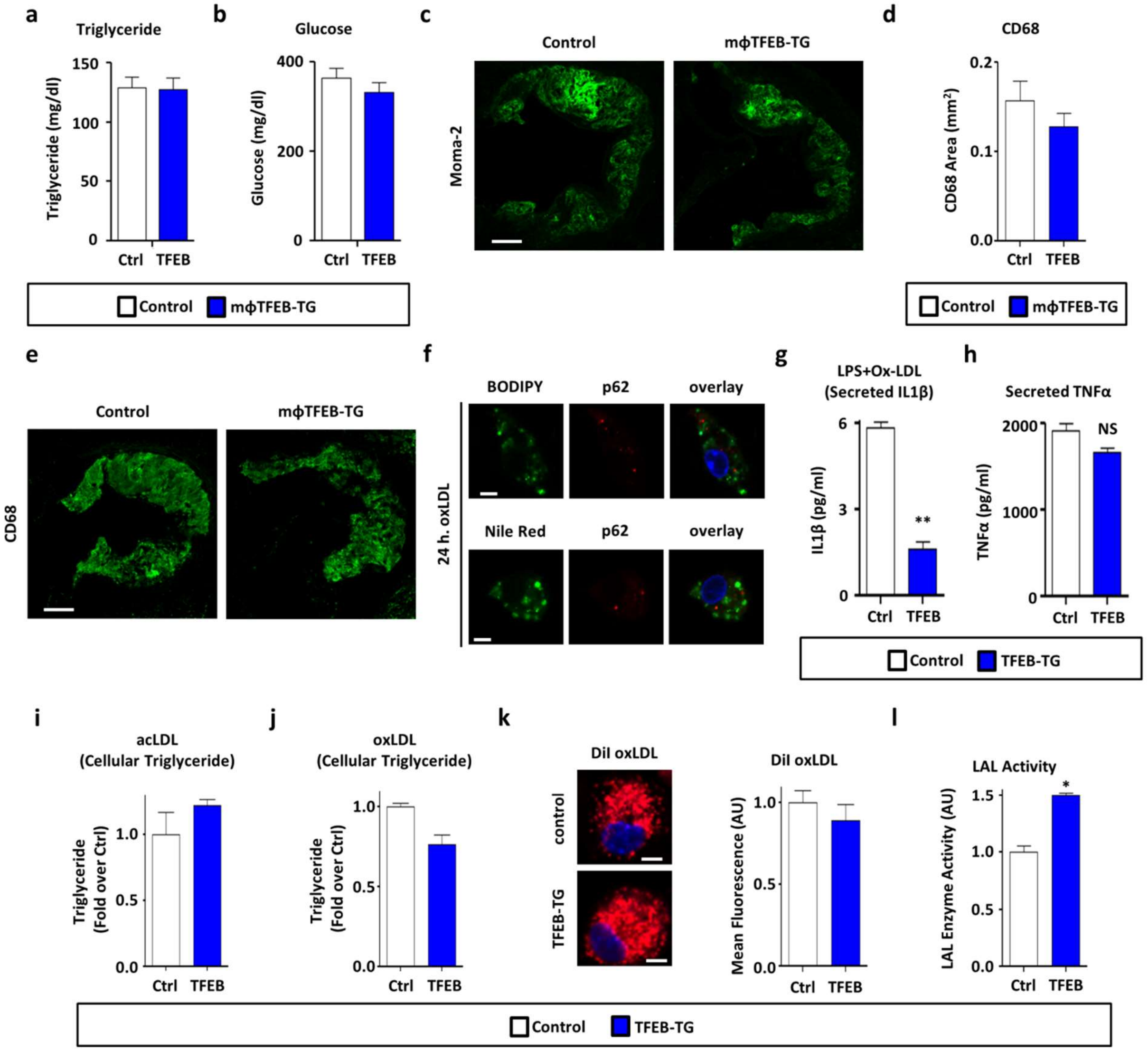

Supplementary Figure 4. Measurements of serum triglycerides **(a)** and glucose **(b)** at two months of Western diet feeding in control and mϕTFEB-TG mice (ApoE-KO background; n≥14 mice per group). **(c)** Representative aortic roots from control and mϕTFEB-TG mice stained with the macrophage marker MOMA-2 (scale bar: 100 μm). **(d, e)** Macrophage content in aortic root sections was analyzed by immunofluorescence staining using an antibody against CD68 (n≥12 mice per group). Representative images are shown in **(e)** (scale bar: 100 μm). **(f)** Immunofluorescence of oxLDL-treated wild-type macrophages co-stained with p62 and lipid markers BODIPY or Nile Red (scale bar: 5 μm). **(g, h)** Control and TFEB-TG macrophages were treated with **(g)** LPS+oxLDL or **(h)** LPS+CC for 24 hours and culture media were assayed for **(g)** IL1β or **(h)** TNFα by ELISA (n=3 independent wells per group). **(i, j)** Graphs represent triglyceride contents of control and TFEB-TG macrophages after incubation with **(i)** acetylated

LDL (acLDL) or **(j)** oxidized LDL (oxLDL) (n=3 independent wells per group). **(k)** Control and TFEB-TG macrophages were treated with DiI-oxidized LDL for 12 hours and intracellular lipid accumulation quantified by immunofluorescence microscopy. (n≥200 cells per group; scale bar: 5 µm) **(l)** Lysosomal acid lipase (LAL) activity from control and TFEB-TG macrophages (n=3 independent wells per group). For all graphs, data are presented as mean ±SEM. *P < 0.05, **P < 0.01, NS=not significant, two-tailed unpaired t-test.

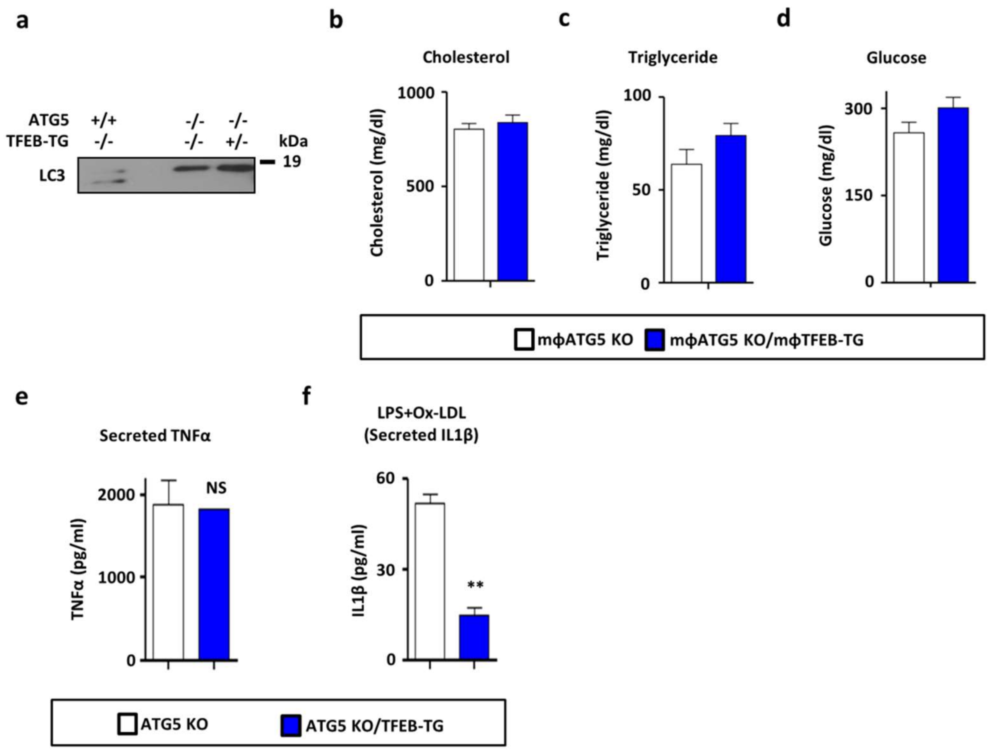

Supplementary Figure 5. (a) Western blot analysis of LC3 from macrophages of the indicated genotypes. **(b-d)** Measurements of serum **(b)** cholesterol, **(c)** triglycerides**,** and **(d)** glucose after two months of Western diet feeding in mϕATG5-KO and dual mϕATG5-KO/mϕTFEB-TG mice (both on ApoE-KO background; n≥11 mice per group). **(e, f)** ATG5-KO and dual ATG5-KO/TFEB-TG macrophages were treated with **(e)** LPS+CC or **(f)** LPS+oxLDL for 24 hours and culture media were assayed for **(e)** TNFα or **(f)** IL1β by ELISA (n=3 independent wells per group). For all graphs, data are presented as mean ±SEM. **P< 0.01, NS=not significant, two-tailed unpaired t-test.

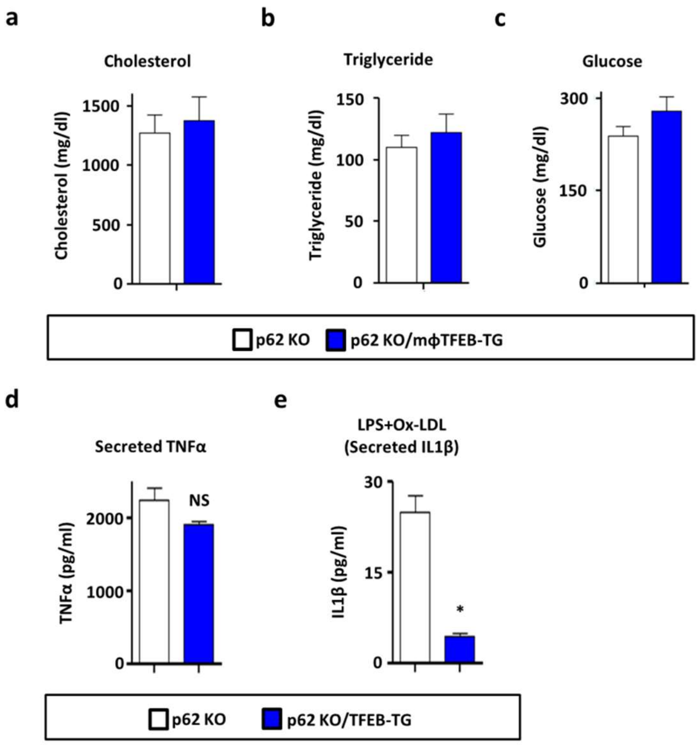

Supplementary Figure 6. Measurements of serum **(a)** cholesterol, **(b)** triglycerides**,** and **(c)** glucose after two months of Western diet feeding in p62-KO and dual p62-KO/mϕTFEB-TG mice (both on ApoE-KO background; n≥11 mice per group). **(d, e)** p62-KO and dual p62-KO/TFEB-TG macrophages were treated with **(d)** LPS+CC or **(e)** LPS+oxLDL for 24 hours and cell culture media were assayed for **(d)** TNFα or **(e)** IL1β by ELISA (n=3 independent wells per group). For all graphs, data are presented as mean ±SEM. *P< 0.05, NS=not significant, two-tailed unpaired t-test.

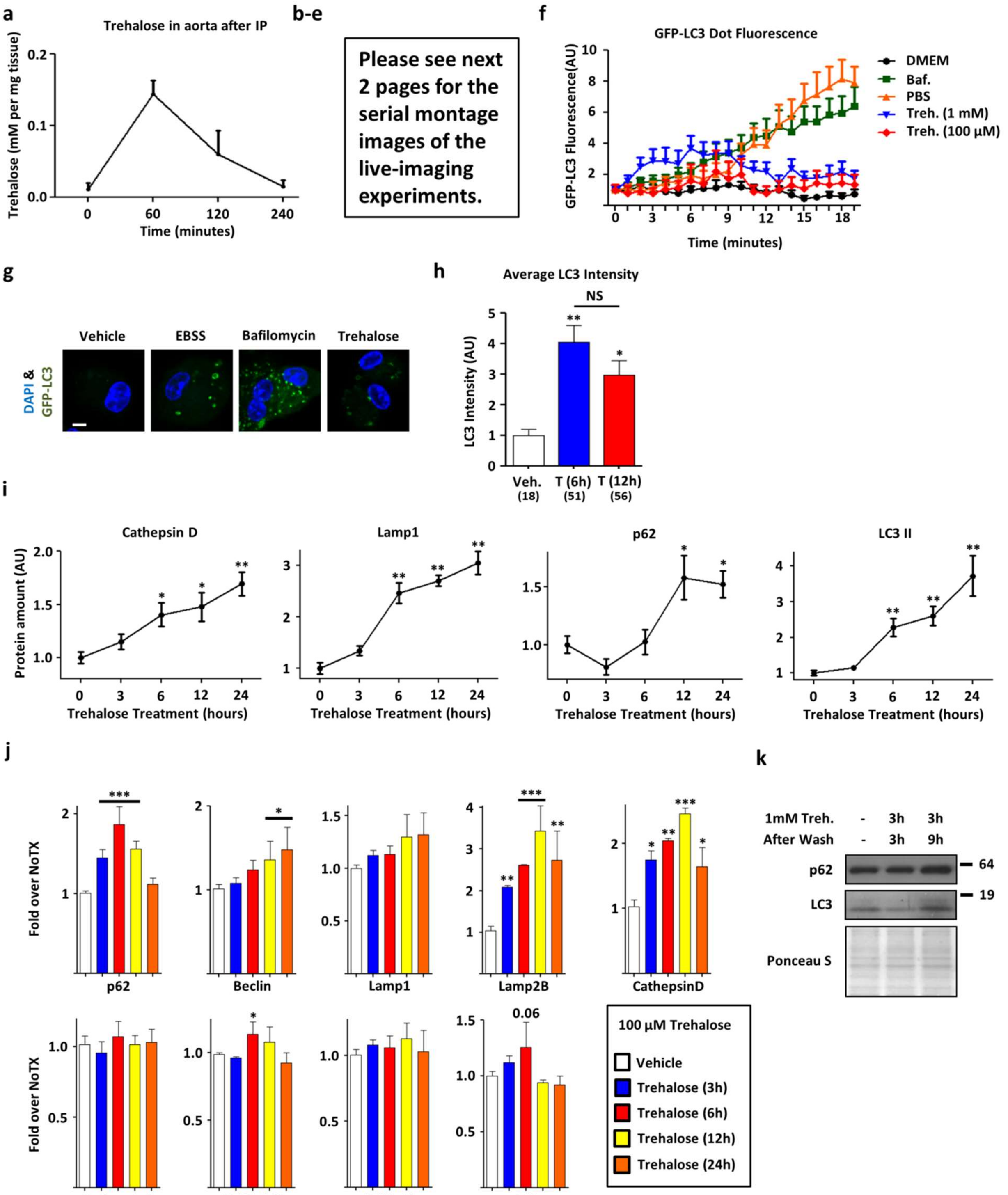
a
Trehalose in aorta after IP
Trehalose (mM per mg tissue)
Time (minutes)
b-e
Please see next 2 pages for the serial montage images of the live-imaging experiments.
f
GFP-LC3 Dot Fluorescence
GFP-LC3 Fluorescence(AU)
Time (minutes)
DMEM
Baf.
PBS
Treh. (1 mM)
Treh. (100 μM)
g
Vehicle
EBSS
Bafilomycin
Trehalose
DAPI & GFP-LC3
h
Average LC3 Intensity
LC3 Intensity (AU)
NS
Veh. (18)
T (6h) (51)
T (12h) (56)
i
Cathepsin D
Lamp1
p62
LC3 II
Protein amount (AU)
Trehalose Treatment (hours)
j
Fold over NoTX
p62
Beclin
Lamp1
Lamp2B
CathepsinD
CathepsinB
ATP6v0d2
ATP6v1h
LC3
0.06
100 μM Trehalose
Vehicle
Trehalose (3h)
Trehalose (6h)
Trehalose (12h)
Trehalose (24h)
k
1mM Treh.
After Wash
3h
9h
p62
LC3
Ponceau S
64
19

b

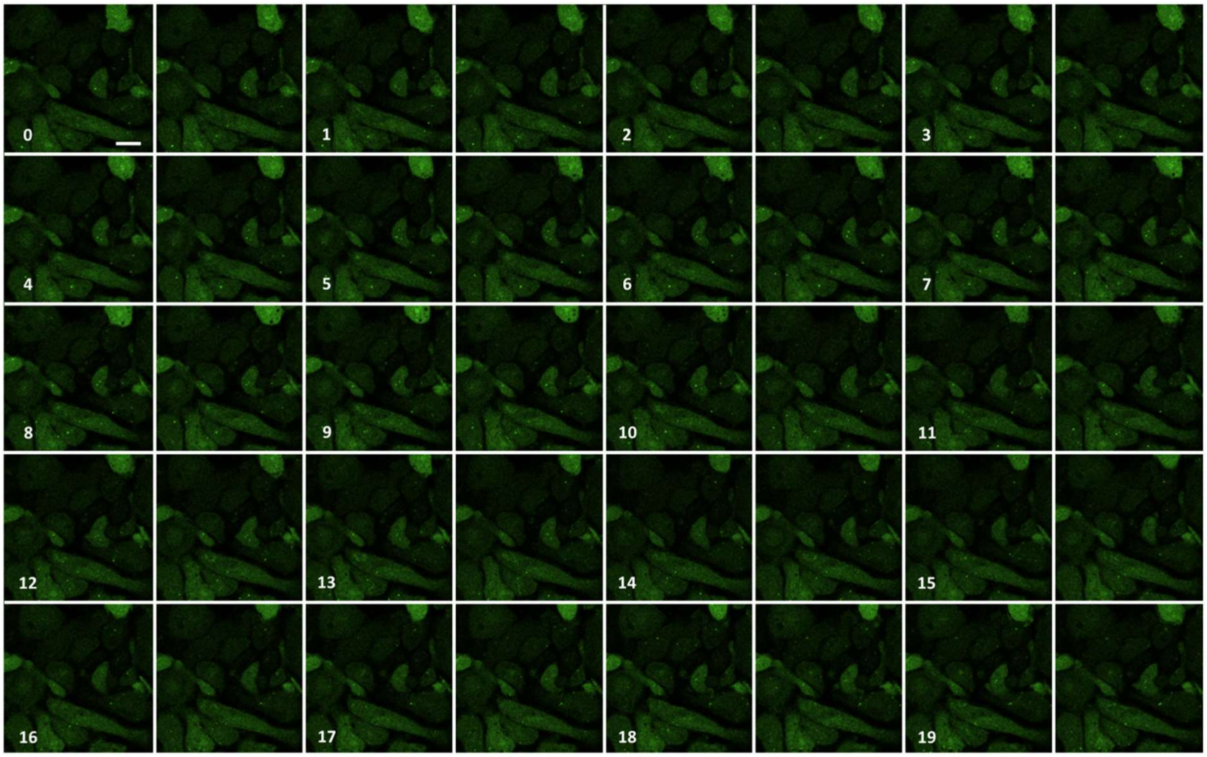

GFP-LC3 peritoneal macrophage in DMEM for 20 minutes (Frame/30 seconds)

c

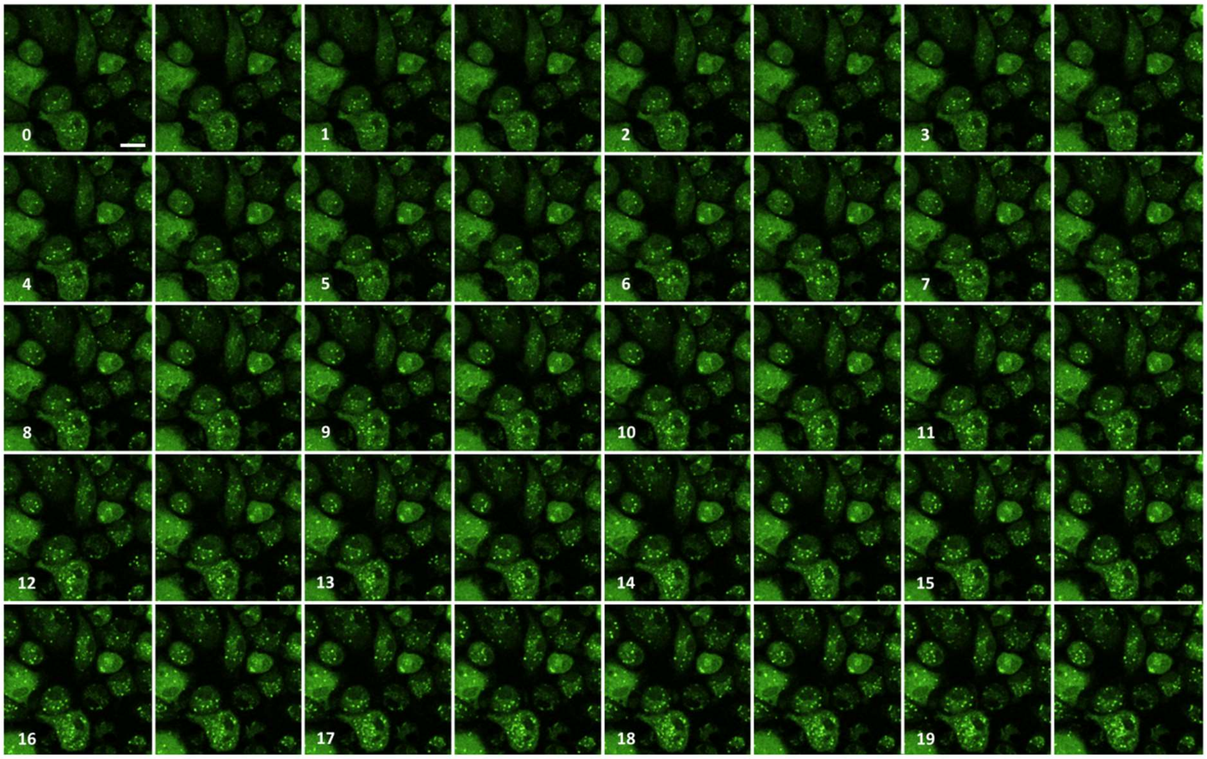

GFP-LC3 peritoneal macrophage in 200 nM Bafilomycin for 20 minutes (Frame/30 seconds)

d

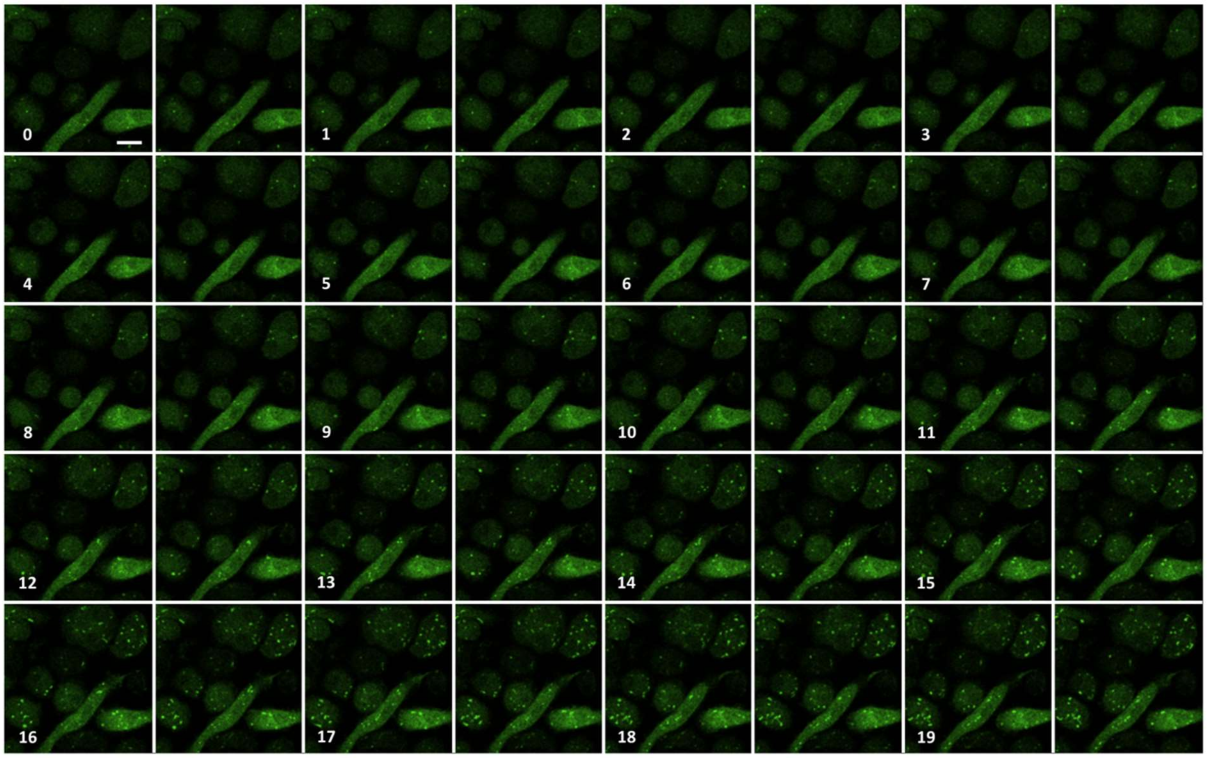

GFP-LC3 peritoneal macrophage in PBS for 20 minutes (Frame/30 seconds)

e

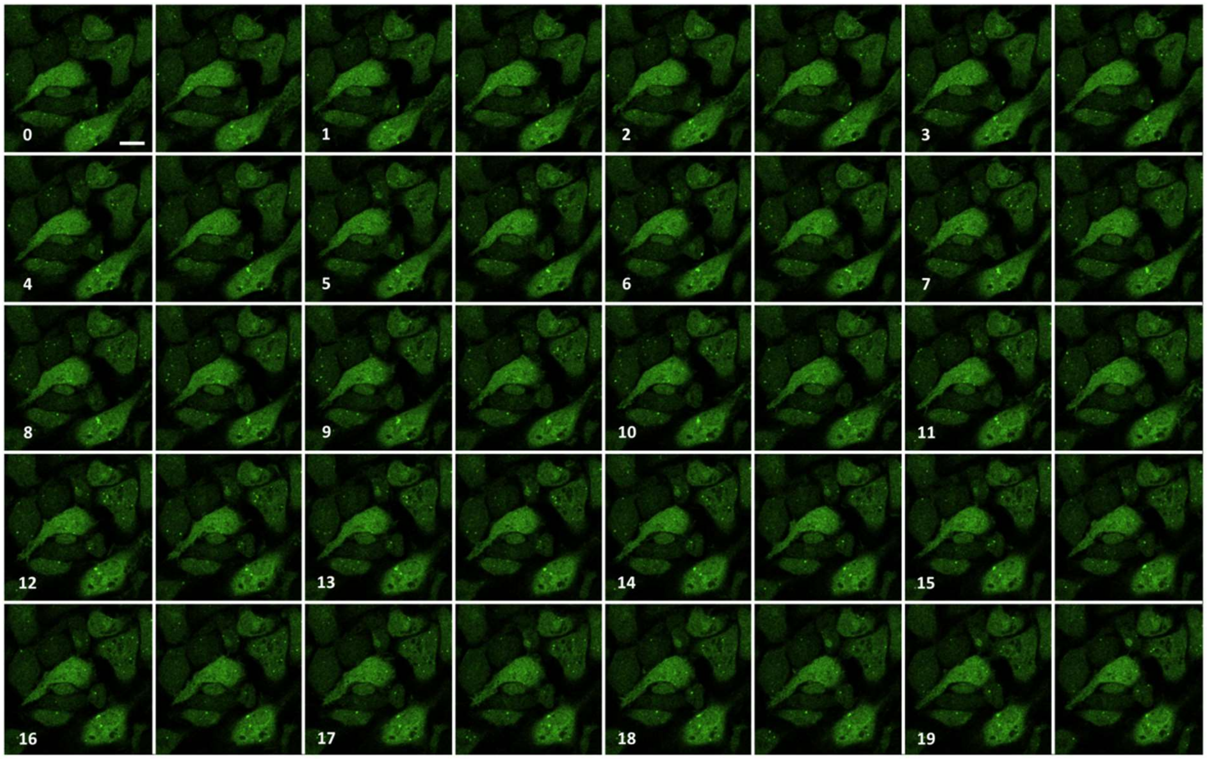

GFP-LC3 peritoneal macrophage in 1mM Trehalose for 20 minutes (Frame/30 seconds)

Supplementary Figure 7. (a) Aortic trehalose levels were measured at the indicated time points from wild-type mice (n≥4) after trehalose injection (3 g/kg, i.p.) by a colorimetric assay. **(b-e)** Representative 20-minute montage of live imaged GFP-LC3 macrophages incubated in **(a)** DMEM, **(b)** 200 nM bafilomycin, **(c)** starvation media (PBS), and **(d)** 1 mM trehalose (scale bar: 10 μm). **(f)** Graph represents GFP dot fluorescence (∑ pixel × fluorescence intensity) of GFP-LC3 expressing macrophages incubated for 20 minutes with the indicated treatments (n≥10 cells for each treatment). **(g)** Representative images of GFP-LC3 expressing macrophages incubated for 30 minutes with indicated treatments (scale bar: 5 μm). **(h)** Graph represents average LC3 staining intensity of wild-type macrophages after trehalose treatment (T) for the indicated times (n is indicated under each bar; statistical significance of differences was calculated using ANOVA followed by Tukey's multiple comparison test). **(i)** Densitometric quantification of Western blots for Cathepsin D, Lamp1, p62, and LC3-II in macrophages treated with trehalose for the indicated times (n=3 independent experiments). Representative blots are shown in Figure 7h. **(j)** Wild-type macrophages were treated with 100 μM trehalose for indicated times and mRNA transcripts of autophagy and lysosome genes were detected by qPCR (n≥3 independent wells for each gene). **(k)** Western blot analysis of p62 and LC3 from the wild-type macrophages incubated with trehalose for 3 hours followed by regular DMEM for the indicated times. Ponceau S staining is shown as loading control. For all graphs, data are presented as mean ±SEM. *P < 0.05, **P < 0.01, ***P < 0.001, NS=not significant, two-tailed unpaired t-test compared to vehicle treatment group except **h**.

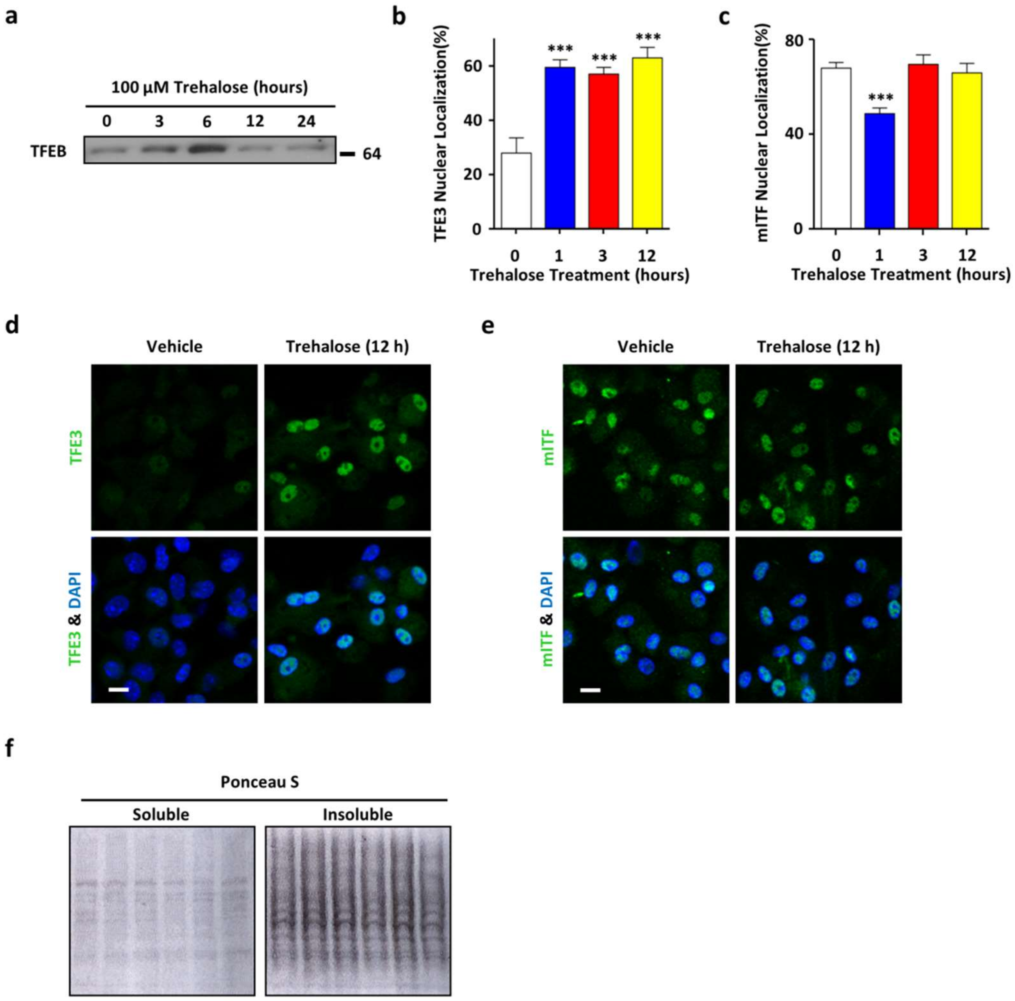

Supplementary Figure 8. (a) Western blot analysis of TFEB in macrophages after 100 μM trehalose treatment for indicated times. **(b, c)** Nuclear localization of **(b)** TFE3 and **(c)** MiTF assessed by immunofluorescence staining after trehalose treatment for the indicated times and quantified as percent cells with nuclear staining (n≥500 cells per group). **(d, e)** Representative images of **(d)** TFE3 and **(e)** and MiTF-stained macrophages after trehalose treatment (scale bar: 10 μm). **(f)** Western blot membranes were stained with Ponceau S and scanned before polyubiquitinated protein antibody (FK-1) incubation to show equal protein loading. Corresponding blots are shown in Figure 8f. For all graphs, data are presented as mean ±SEM. ***P < 0.001, two-tailed unpaired t-test compared to vehicle treatment group.

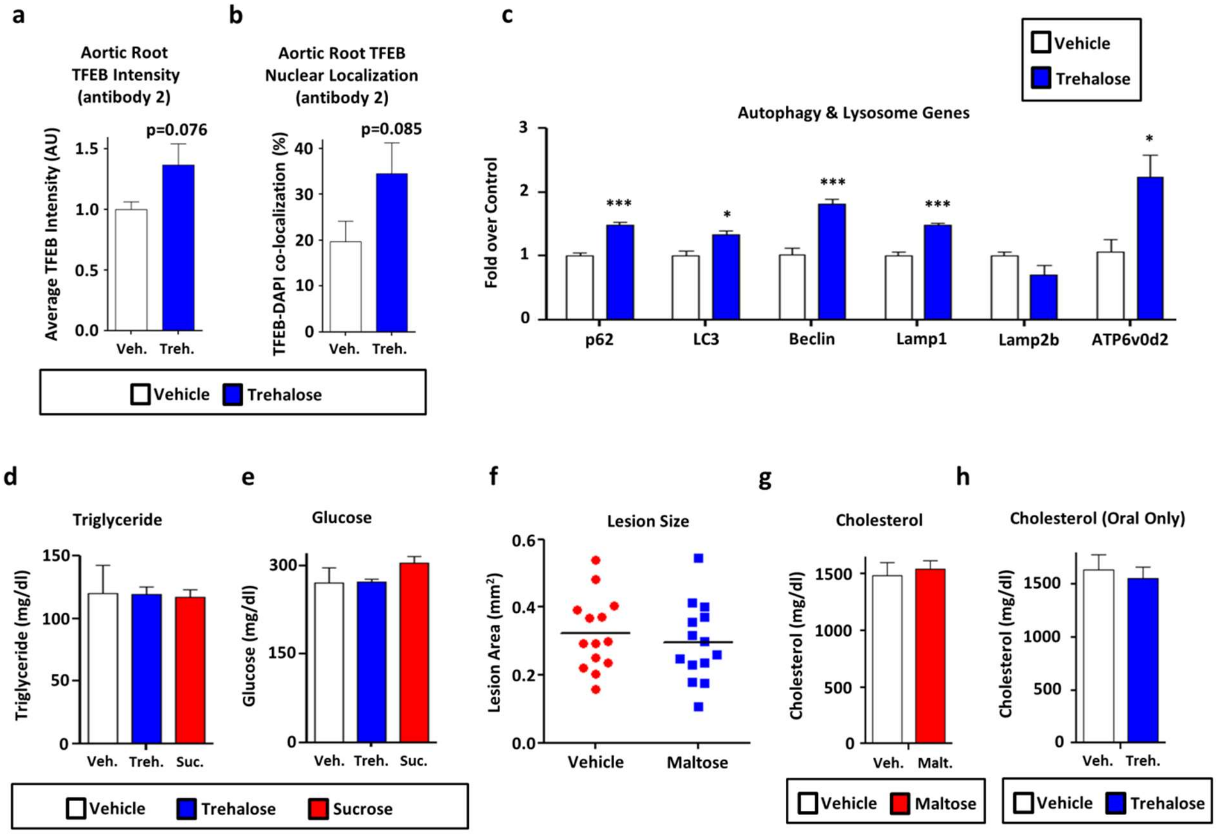

Supplementary Figure 9. (a,b) TFEB intensity and nuclear localization in aortic roots of the same cohort used in Figure 9a-e was detected by immunofluorescence using a different TFEB antibody. Shown are **(a)** average aortic root TFEB intensity and **(b)** TFEB-DAPI co-localization (n=4 mice per group). **(c)** Cohorts of wild-type mice were administered vehicle or trehalose (2 g/kg given 5x/week i.p. for 2 weeks, n=4 mice per group), splenic macrophages were isolated, and autophagy-lysosome markers evaluated by qPCR. **(d, e)** Measurements of serum triglycerides **(d)** and glucose **(e)** at two months of Western diet feeding in vehicle-, trehalose- or sucrose- injected ApoE-KO mice (n≥7 mice per group). **(f)** A cohort of ApoE-KO mice were fed a Western diet for 2 months while being administered either vehicle or maltose (2 g/kg maltose given 3x/week i.p. and 3% maltose given *ad lib* in drinking water). Graphs represent quantification of Oil Red O-stained atherosclerotic plaques at the level of aortic root (Statistical significance of differences was calculated using Mann-Whitney U test, not significant). **(g, h)** Measurements of serum cholesterol at two months of Western diet feeding in ApoE-KO mice either maltose-injected **(**n≥13 mice per group, **g)** or oral trehalose-supplemented **(**n≥9 mice per group, **h)** cohorts. For all graphs, data are presented as mean ±SEM. *P< 0.05, ***P < 0.001, two-tailed unpaired t-test except **f**.

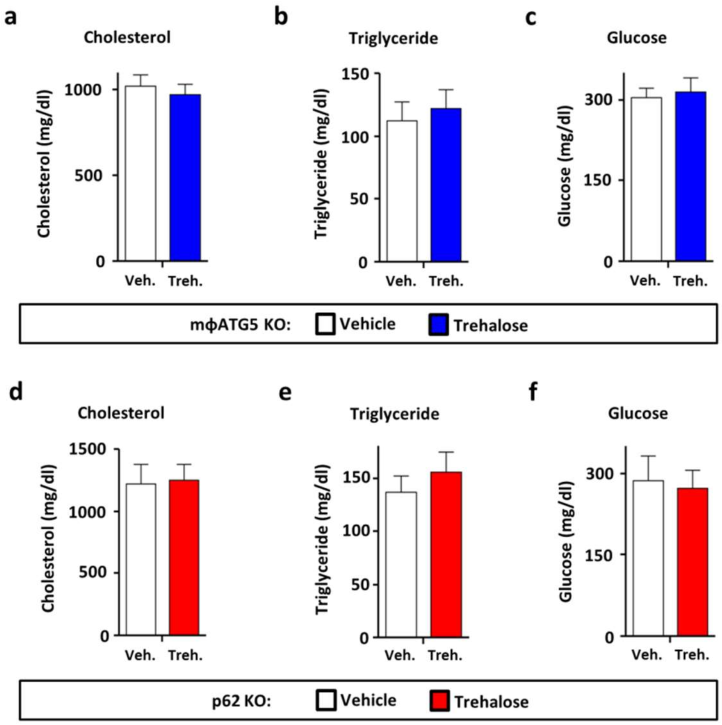

Supplementary Figure 10. Measurements of serum cholesterol **(a, d)**, triglycerides **(b, e)** and glucose **(c, f)** at two months of Western diet feeding in vehicle or trehalose injected mice cohorts (both in mϕATG5-KO and ApoE-KO background **(a-c)**; n≥11 mice per group or in p62-KO and ApoE-KO background **(d-f)**; n≥6 mice per group). For all graphs, data are presented as mean ±SEM, two-tailed unpaired t-test.

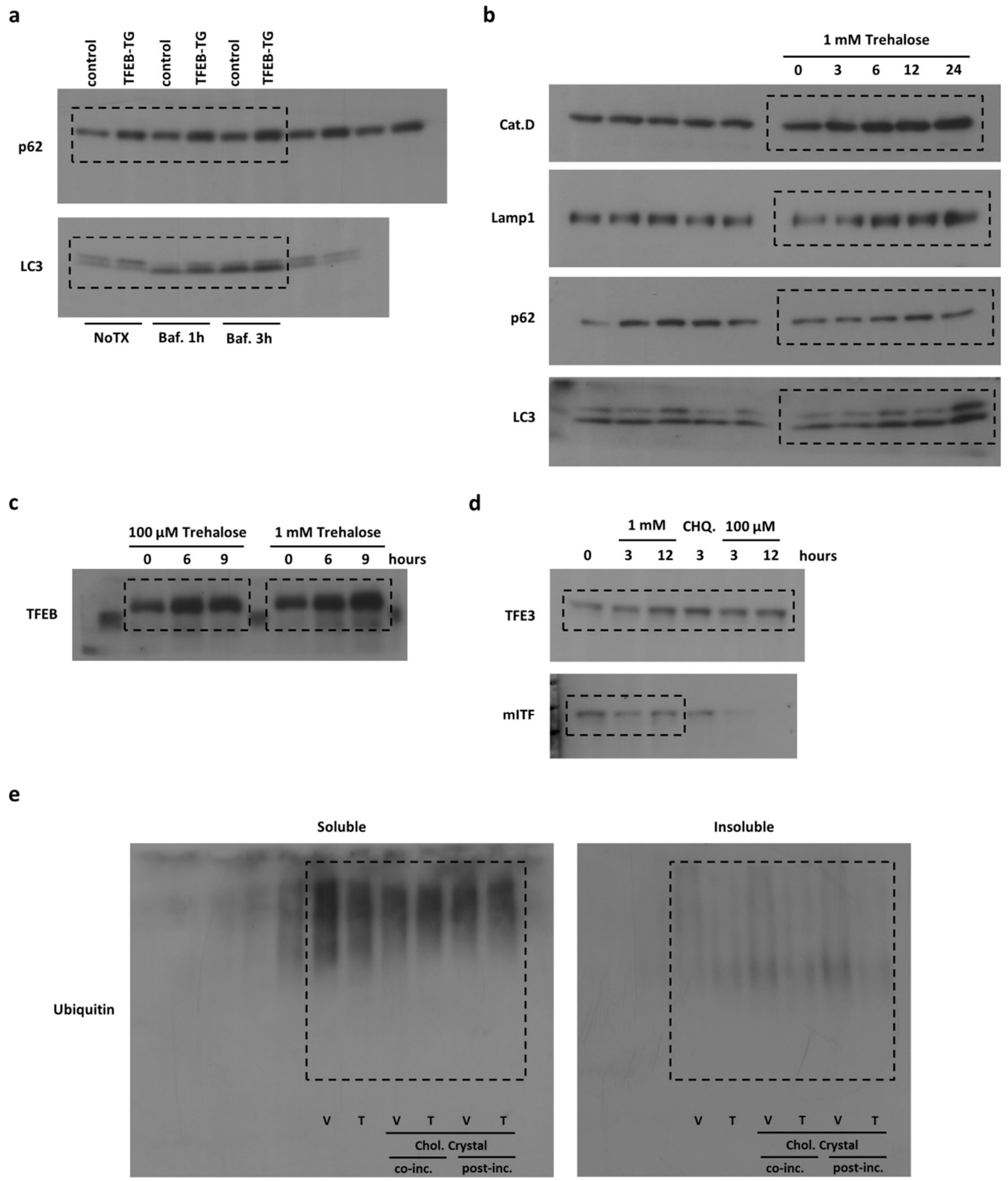

Supplementary Figure 11. Uncropped western blot scans used in the main figures. Blots from Figure 2d **(a)**, Figure 7h **(b)**, Figure 8a **(c)**, Figure 8c **(d)**, and Figure 8f **(e)**.

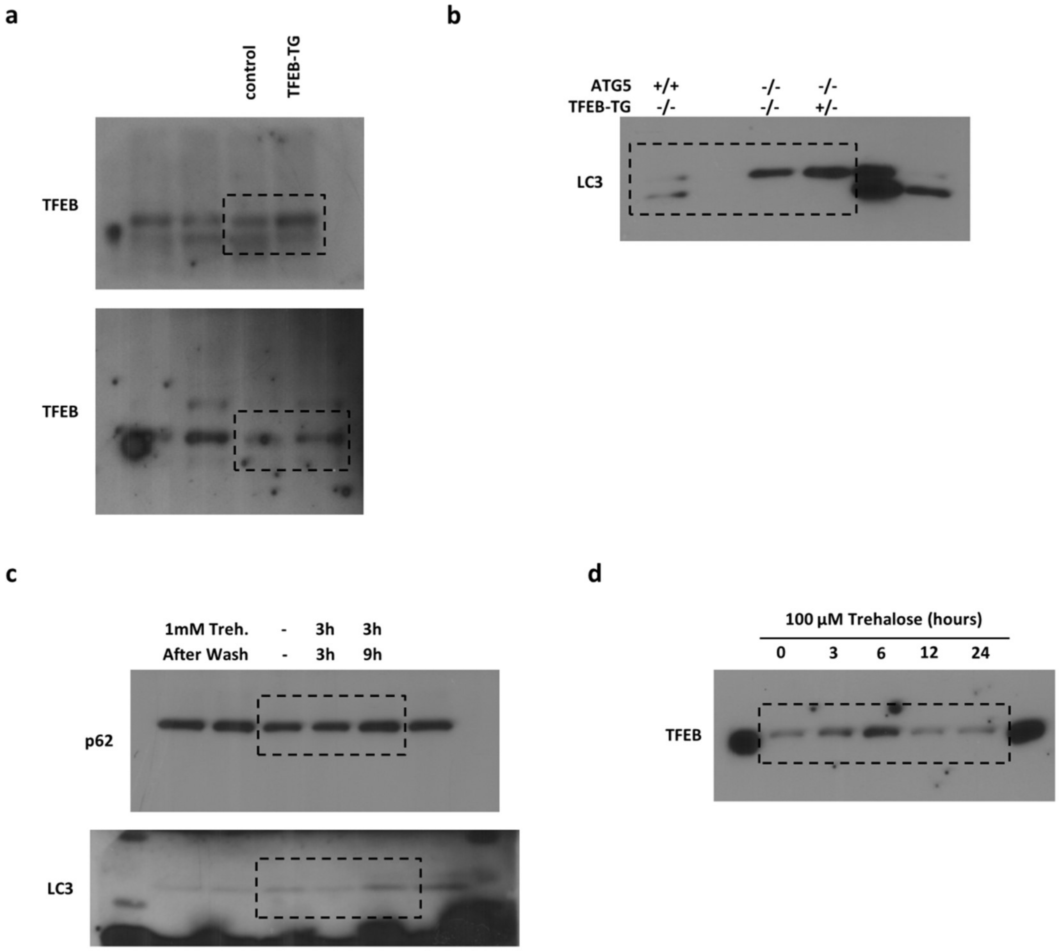

Supplementary Figure 12. Uncropped western blot scans used in the supplementary figures. Blots from Supplementary Figure 2a **(a)**, Supplementary Figure 5a **(b)**, Supplementary Figure 7k **(c)**, and Figure 8a **(d)**.

Genotype	Primers	Bands
TFEB-TG	5’-CAT CAA CCC TGA GAT GCA GAT GCC TAA CAC<br>5’-TGT GAT TGT CTT TCT TCT GCC GCT CCT TGG	WT: No Band<br>TG: ~200
p62 KO	5’-CTG CAT GTC TTC TCC CAT GAC<br>5’-TAG ATA CCT AGG TGA GCT CTG<br>5’-CCT ACG GGT CCT TTT CCC AAC<br>5’-TCC TCC TTG CCC AGA AGA TAG	WT band: ~300 bp<br>KO band: ~600 bp
ATG5 Floxed	5’-GTA CTG CAT AAT GGT TTA ACT CTT GCT AGA<br>5’-GTT TGA ATA TGA AGG CAC ACC CCT GAA ATG<br>5’-GTG CAA TCC ATC TTG TTC AAT GGC CGA TCC	WT band:~450 bp<br>Floxed band:~350 bp
Cre	5’-GCA TTA CCG GTC GAT GCA ACG AGT GAT GAG<br>5’-GAG TGA ACG AAC CTG GTC GAA ATC AGT GCG	WT: No band<br>Cre: ~400
GFP-LC3 TG	5’-TCC TGC TGG AGT TCG TGA CCG<br>5’-TTG CGA ATT CTC AGC CGT CTT CAT CTC TCT CGC	WT: No band<br>TG: ~400 bp
ApoE KO	5’-GCC TAG CCG AGG GAG AGC CG<br>5’-TGT GAG TTG GGA GCT CTG CAG C<br>5’-GCC GCC CCG ACT GCA TCT	WT band: ~150 bp<br>KO band: ~250 bp

Supplementary Table 1. Genotyping Primer Sequences

Primer	Direction	Sequence
Atp6v0d2	F	5’- CAG AGC TGT ACT TCA ATG TGG AC -3’
Atp6v0d2	R	5’- AGG TCT CAC ACT GCA CTA GGT -3’
Atp6v1h	F	5’- GGA TGC TGC TGT CCC AAC TAA -3’
Atp6v1h	R	5’- TCT CTT GCT TGT CCT CGG AAC -3’
Beclin	F	5’- AAT CTA AGG AGT TGC CGT TAT AC -3’
Beclin	R	5’- CCA GTG TCT TCA ATC TTG CC -3’
Cathepsin B	F	5’- TCC TTG ATC CTT CTT TCT TGC C -3’
Cathepsin B	R	5’- ACA GTG CCA CAC AGC TTC TTC -3’
Cathepsin D	F	5’- GCT TCC GGT CTT TGA CAA CCT -3’
Cathepsin D	R	5’- CAC CAA GCA TTA GTT CTC CTC C -3’
Lamp1	F	5’- ACA TCA GCC CAA ATG ACA CA -3’
Lamp1	R	5’- GGC TAG AGC TGG CAT TCA TC -3’
Lamp2b	F	5’- GGT GCT GGT CTT TCA GGC TTG ATT -3’
Lamp2b	R	5’- ACC ACC CAA TCT AAG AGC AGG ACT -3’
LC3	F	5’- CGT CCT GGA CAA GAC CAA GT -3’
LC3	R	5’-ATT GCT GTC CCG AAT GTC TC -3’
p62	F	5’- GCT GCC CTA TAC CCA CAT CT -3’
p62	R	5’-CGC CTT CAT CCG AGA AAC -3’
TFEB	F	5’- GGT GCA GTC CTA CCT GGA GA -3’
TFEB	R	5’- GTG GGC AGC AAA CTT GTT CC -3’

Supplementary Table 2. RT-PCR Primer Sequences
